# Upregulation of a Circular *BAX* Transcript in Breast Cancer Is Associated with Unfavorable Prognosis

**DOI:** 10.3390/ijms27104160

**Published:** 2026-05-07

**Authors:** Vaia K. Stafyla, Spyridon Christodoulou, Panagiotis Tsiakanikas, Nikolaos Michalopoulos, Nikolaos Danias, Panagiotis Kokoropoulos, Christos K. Kontos, Nikolaos Arkadopoulos

**Affiliations:** 1 Fourth Department of Surgery, University General Hospital “Attikon”, National and Kapodistrian University of Athens, 12462 Athens, Greece; vaniastafyla07@gmail.com (V.K.S.); spyridon.christodoulou@yahoo.gr (S.C.); ndanias@med.uoa.gr (N.D.); kokoropoulos@yahoo.gr (P.K.); narkado@hotmail.com (N.A.); 2 Department of Biochemistry and Molecular Biology, Faculty of Biology, National and Kapodistrian University of Athens, Panepistimiopolis, 15771 Athens, Greece; ptsiak@biol.uoa.gr; 3 First Department of Propaedeutic Surgery, “Hippokration” General Hospital of Athens, National and Kapodistrian University of Athens, 11527 Athens, Greece; nmichal@med.uoa.gr

**Keywords:** breast malignancy, circular RNA (circRNA), non-coding RNA, cancer biomarker, prognostic biomarker, tumor recurrence

## Abstract

Recently, we have discovered several alternative circRNAs produced by alternative circularization of the primary transcripts of the apoptosis-related BAX gene. Among them, only three circRNAs, namely circ-BAX-6c, circ-BAX-18 and circ-BAX-74, are expressed in all studied triple-negative breast cancer (BrCa) cell lines and in the normal one; moreover, circ-BAX-18 is the only one comprising a novel microexon with canonical splice sites. Therefore, in this study, we examined circ-BAX-18 expression in BrCa tissues and its potential association with tumor features and patients’ prognosis. For this purpose, tumor samples from a cohort of 144 female BrCa patients were used, along with paired non-cancerous breast tissue for 14 cases. circ-BAX-18 expression levels were quantified using an optimized, *in-house*-developed quantitative real-time PCR assay. Extensive biostatistical analysis was performed, including survival analysis. The expression of circ-BAX-18 differed among the 14 pairs of normal and cancerous breast tissues (*p* = 0.002). On the other hand, circ-BAX-18 expression did not appear to be associated with any clinicopathological characteristics. After splitting at the median value, higher circ-BAX-18 expression was rather associated with poorer disease-free survival (*p* = 0.009) and overall survival (*p* = 0.012) of BrCa patients. According to the multivariate Cox regression analysis results, the prognostic value of circ-BAX-18 positivity was rather independent of the other established predictors of prognosis incorporated in the Cox regression models, such as the molecular subtype of the tumors and the prognostic stage of the disease (regarding DFS: HR = 2.53, 95% CI = 1.30–4.92, *p* = 0.006; regarding OS: HR = 2.32, 95% CI = 1.20–4.49, *p* = 0.012). Moreover, the stratification of patients based on prognostic staging showed that high circ-BAX-18 expression may distinguish among stage II patients those with worse prognosis. In conclusion, increased circ-BAX-18 expression in BrCa is most likely associated with poor prognostic outcomes.

## 1. Introduction

The World Health Organization (WHO) reports that breast cancer (BrCa) is the primary cause of cancer-related mortality among women worldwide, accounting for approximately 720,000 deaths and 2.5 million new cases each year. The disease outlook is concerning, as both the incidence and mortality rates are expected to increase by roughly 20% by 2035. Successful clinical management of BrCa relies heavily on early diagnosis, with digital mammography remaining the gold standard screening modality [1]. The five-year survival rate for patients with localized disease exceeds 95%, while this rate declines to 33% for those with distant metastasis. Invasive BrCa of no special type (NST) is the most common histological entity, representing up to 80% of all BrCa cases. However, this broad category comprises a highly heterogeneous group of tumors characterized by distinct morphological and clinical features [2,3].

Anatomic staging using the Tumor, Node, Metastasis (TNM) system provides fundamental prognostic information; however, recent guideline amendments incorporate molecular characteristics, such as the expression status of estrogen and progesterone receptors (ER and PR), human epidermal growth factor receptor 2 (HER2), the Ki-67 index and gene expression profiling, to formulate a parallel, more accurate prognostic staging system [4]. Based on these markers, breast tumors are classified into four primary molecular subtypes: Luminal A is the most common subtype, characterized by high hormone receptor expression and low proliferation. Consequently, these tumors respond well to endocrine therapy and generally have excellent prognosis. In contrast, luminal B tumors express lower levels of hormone receptors, have enhanced proliferation and occasionally present HER2 co-amplification. These features render luminal B less responsive to endocrine therapy and are consistent with more aggressive phenotypes prone to higher relapse rates [5]. Breast tumors that overexpress HER2—resulting from erb-b2 receptor tyrosine kinase 2 (*ERBB2*) amplification—were once linked to a poor prognosis but now can benefit significantly from targeted therapies, which improve patient outcomes [6]. Finally, triple-negative BrCa lacks ER, PR, and HER2 expression and is frequently associated with *BRCA* mutations and early-onset disease. TNBC is an aggressive subtype characterized by frequent recurrence, significant metastatic potential and poor long-term survival [7,8].

Circular RNAs (circRNAs) are a unique class of RNA molecules forming a covalent ring structure resulting from the back-splicing of pre-mRNAs. They exhibit broad expression patterns across various tissues and cell lines. Although their functional roles are still being elucidated, circRNAs are broadly expressed across tissues and implicated in the modulation of gene expression through diverse mechanisms. These include microRNA (miRNA) sponging, interacting with RNA-binding proteins and regulating transcription through direct binding on RNA polymerase II or transcription factors, while some circRNAs are also reported to encode peptides [9]. Aberrant circRNA expression is well-documented in human malignancies, particularly in BrCa [10,11]. In BrCa, circRNAs can function as both promoters of tumorigenesis and tumor suppressors, being implicated in programmed cell death (apoptosis), metabolic reprogramming, resistance to therapy, invasion and metastasis [12,13,14,15,16,17]. These properties, together with their remarkable stability in bodily fluids, highlight the translational potential of circRNAs as biomarkers for non-invasive disease monitoring and risk stratification [18].

Apoptosis is a major type of regulated cell death in multicellular organisms, which is activated by cellular stress and/or damage to maintain tissue homeostasis [19]. Typical signals that trigger the apoptotic cascade include the mitochondrial release of cytochrome c, diablo IAP-binding mitochondrial protein (DIABLO) and/or the activation of cell surface death receptors. These events disrupt the equilibrium between pro- and anti-apoptotic factors, leading to the subsequent activation of cysteine-dependent proteases known as caspases, which are the primary mediators of apoptosis [19,20]. In BrCa, the impairment of apoptosis-related genes skews the balance towards uncontrolled proliferation, promoting tumor development and resistance to therapy [21].

The human BCL2-associated X (BAX) gene, located on chromosome 19, consists of seven exons and plays a key role as an activator of the intrinsic apoptotic pathway. It encodes a pro-apoptotic effector protein containing three Bcl-2 homology domains (BH1–BH3). Upon activation by BH3-only proteins, BAX monomers translocate to the mitochondrial outer membrane and disrupt membrane integrity, facilitating the release of cytochrome c into the cytosol [22,23]. Multiple studies have shown that *BAX* gene and protein expression levels are key indicators of pro-apoptotic signaling, linked to better prognosis and improved response to treatment in BrCa [24,25,26,27]. Furthermore, the *BAX* gene undergoes extensive alternative splicing, which leads to the overexpression of specific transcript variants in breast tumors [28]. This extensive alternative splicing, along with alternative circularization (back-splicing), results in a plethora of *BAX*-derived circRNAs, some of which have previously been detected in chronic lymphocytic leukemia (CLL) [29]. Recently, using nanopore sequencing, our research group expanded this pool by identifying additional, novel *BAX*-derived circRNAs in BrCa cell lines [30]. Despite their broad expression and their potential to modulate host gene mRNA expression during carcinogenesis, the exact functional roles of these circRNAs and their involvement in cancer pathophysiology remain unexplored.

Among these newly identified circRNAs, only three, namely circ-BAX-6c, circ-BAX-18 and circ-BAX-74, are expressed in all studied triple-negative breast cancer (BrCa) cell lines and in the normal one; moreover, circ-BAX-18 is the only one comprising a novel microexon with canonical splice sites, located between *BAX* exons 4 and 5. In linear transcripts, inclusion of such microexons can generate premature termination codons that trigger nonsense-mediated decay [31]. However, in most cases, open reading frames are preserved, resulting in an altered amino acid sequence and structure of nascent protein isoforms; this process is often linked to cancer and neurodevelopmental disorders [32]. Interestingly, microexons are preferentially retained in circRNAs, as circular transcripts are generally not translated and thus escape from the aforementioned RNA surveillance mechanism [31,33]. The presence of microexons expands the functional repertoire of these circRNAs, potentially acting as transcriptional regulators or particular binding sites for miRNAs and/or RNA-binding proteins [31]. Considering its distinctive structural features and broad expression profile in BrCa cell lines, we selected circ-BAX-18 as our primary target for this study. Therefore, in this study, we examined its expression in BrCa tissues and evaluated its potential association with tumor features and BrCa patients’ prognosis.

## 2. Results

### 2.1. Clinicopathological Characteristics of Female BrCa Patients

The cohort of female patients with primary BrCa comprised 144 cancerous tissue specimens, while in 14 cases paired non-cancerous breast tissue specimens were also available. The patients’ median age was 60 years at the time of diagnosis (range: 32–90 years). The histological grade was assessed according to the World Health Organization (WHO) classification system; 7 patients were diagnosed with grade I (well-differentiated), 93 with grade II (moderately differentiated), and 44 with grade III malignant tumors (poorly differentiated). Additionally, based on the TNM stage classification, 40 malignant breast tumors were designated as stage I (27.8%), 86 as stage II (59.7%) and 18 as stage III (12.5%). The biological and clinicopathological features of the BrCa patients of this study are summarized in Table 1.

### 2.2. Underexpression of circ-BAX-18 in BrCa Tissues Compared to Their Non-Cancerous Counterparts

**Figure 1:**
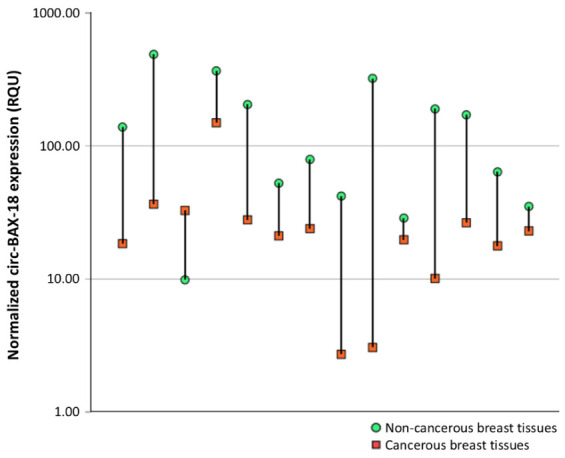
Comparison of circ-BAX-18 levels in 14 cancerous breast tissues and their non-malignant counterparts. The Wilcoxon signed-rank statistical test was applied to calculate the *p* value.

The comparison of circ-BAX-18 levels among 14 pairs of malignant breast tumors and their non-cancerous breast tissue counterparts revealed a profound decrease of the circ-BAX-18 expression in the vast majority of malignant breast tumors (*p* = 0.002) (Figure 1). However, the distribution of expression levels in the normal samples and the total number of cancer samples exhibited a remarkable overlap. Thus, the median value of circ-BAX-18 expression in the 144 BrCa samples was 29.78 ± 2.43 RQU, with a range of 0.63 to 149.4 and an interquartile range of 8.51–40.34 RQU. The respective values quantified in the 14 normal tissue samples are presented in detail in Table 2.

In order to examine the existence of any association between circ-BAX18 expression and each clinicopathological feature of the tumors, we decided to split at the median value in order to classify circ-BAX-18 expression in each tissue specimen as positive or negative; the same cut-off value (20.25 RQU) was needed for the survival analysis performed thereafter, as explained in the “Materials and Methods” Section. As a result, 72 (50%) samples were classified as circ-BAX-18-negative and another 72 (50%) samples as circ-BAX-18-positive.

Any potential association of the circ-BAX-18 expression status with tumor features such as the molecular subtype, anatomic stage, prognostic stage, mitotic rate, HER2, ER, and PR status was then examined. circ-BAX-18 expression was not significantly correlated with any of these parameters.

### 2.3. circ-BAX-18 Overexpression Predicts Poor Prognosis in BrCa Patients, Independently of Other Clinicopathological Factors

The survival analysis we performed included Kaplan–Meier analysis and Cox regression, regarding both the disease-free survival (DFS) and overall survival (OS). Particularly in Cox regression, bootstrapping based on 1000 samples was applied to strengthen any conclusions. Survival data were recorded in 140 BrCa cases. The median follow-up time of patients was 92 months.

**Figure 2:**
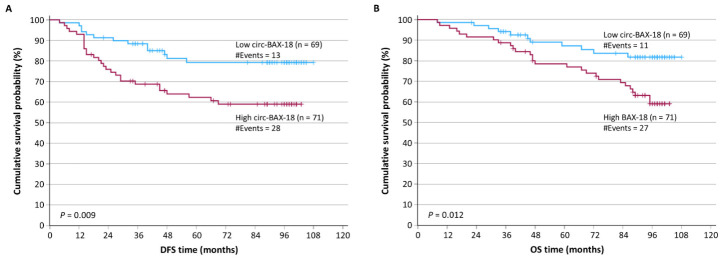
Kaplan–Meier survival analysis for the DFS (**A**) and OS (**B**) of BrCa patients, stratified based on circ-BAX-18 expression status. Overexpression of circ-BAX-18 was an unfavorable prognosticator. The *p* value was calculated using the log-rank (Mantel–Cox) test.

Kaplan–Meier DFS curves depicted that circ-BAX-18-positive BrCa patients were more likely to present with a relapse, compared to patients with circ-BAX-18-negative breast tumors (*p* = 0.009) (Figure 2A). The prognostic significance of circ-BAX-18 expression regarding patients’ DFS was supported by the findings of the univariate Cox regression analysis. BrCa patients with circ-BAX-18-positive expression status were shown to entail a higher risk of relapse, in comparison with circ-BAX-18-negative ones (HR = 2.32, 95% CI = 1.20–4.49, *p* = 0.012, BCa bootstrap 95% CI = 1.19–4.94, bootstrap *p* = 0.006). Besides that, the molecular subtype and prognostic stage of the disease were also significant indicators of prognosis with regard to DFS (*p* < 0.001 for both these variables). Thus, the multivariate Cox regression analysis included the circ-BAX-18 expression status and the prognostic stage (Table 3), or alternatively the circ-BAX-18 expression status, anatomic stage, and molecular subtype (Table S1). Interestingly, a positive circ-BAX-18 expression status was shown to be an independent and strong predictor of tumor recurrence (HR = 2.53, 95% CI = 1.30–4.92, *p* = 0.006, BCa bootstrap 95% CI = 1.19–6.94, bootstrap *p* = 0.003).

Kaplan–Meier OS analysis demonstrated that circ-BAX-18-positive BrCa patients were more likely to succumb to their disease, compared to patients with circ-BAX-18-negative breast tumors (*p* = 0.012) (Figure 2B). The prognostic significance of circ-BAX-18 expression regarding patients’ OS was also supported by the results of univariate Cox regression analysis. Particularly, we observed that BrCa patients with circ-BAX-18-positive expression status entailed a higher risk of disease-related death as compared to circ-BAX-18-negative ones (HR = 2.38, 95% CI = 1.18–4.81, *p* = 0.015, BCa bootstrap 95% CI = 1.31–5.71, bootstrap *p* = 0.010). Furthermore, the molecular subtype and prognostic stage proved to be very significant prognosticators of OS. Thus, the multivariate Cox regression analysis included the circ-BAX-18 expression status and the prognostic stage (Table 4), or alternatively the circ-BAX-18 expression status, anatomic stage, and molecular subtype (Table S2). Interestingly, after performing bootstrapping, BrCa patients bearing circ-BAX-18-positive tumors had a significantly shorter OS time interval than those with inferior circ-BAX-18 levels (HR = 2.50, 95% CI = 1.24–5.04, *p* = 0.011, BCa bootstrap 95% CI = 1.16–7.24, bootstrap *p* = 0.015), independently of the molecular subtype of the breast tumor and the prognostic stage of the disease. Apparently, circ-BAX-18 positivity in BrCa may hence be considered as a novel independent unfavorable predictor of OS.

### 2.4. Prognostic Value of circ-BAX-18 Expression in BrCa Patients, Stratified According to Molecular Subtype, Anatomic Stage, or Prognostic Stage

**Figure 3:**
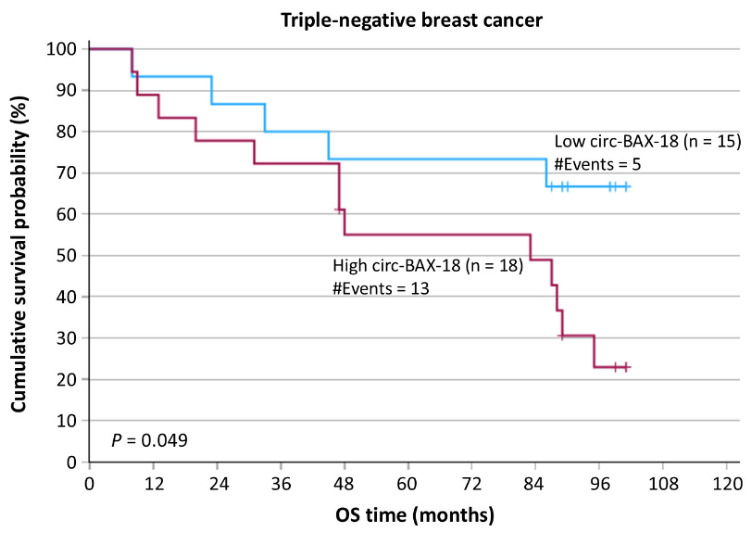
Stratified Kaplan–Meier survival curves for the OS of triple-negative BrCa patients. Patients with triple-negative breast tumors being positive for circ-BAX-18 expression had shorter OS time intervals than patients with circ-BAX-18-negative triple-negative breast tumors. The *p* value was calculated using the log-rank (Mantel–Cox) test.

Features of breast neoplasms such as molecular subtype and tumor grade are pivotal for the prognosis of BrCa patients. For this reason, we stratified patients according to these variables so as to further assess the potential additional impact of circ-BAX-18 expression status in each subgroup. The stratification according to molecular subtype showed that patients with triple-negative or HER2-positive tumors had significantly lower DFS and OS rates, in comparison with those with luminal A or luminal B breast tumors (Figure S1), as might be expected. Interestingly, triple-negative BrCa patients positive for circ-BAX-18 expression exhibited a trend for inferior survival rates, as compared to those bearing circ-BAX-18-negative triple-negative tumors (*p* = 0.049) (Figure 3).

**Figure 4:**
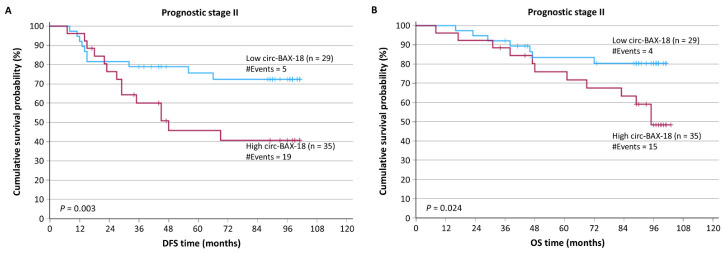
Stratified Kaplan–Meier survival curves for the DFS and OS of BrCa patients, according to the prognostic stage. Patients with circ-BAX-18-positive tumors of prognostic stage II had shorter DFS (**A**) and OS (**B**) time intervals than those with circ-BAX-18-negative tumors. The *p* values were calculated using the log-rank (Mantel–Cox) test.

The stratification of patients according to prognostic stage revealed that patients of prognostic stage III had significantly shorter DFS and OS intervals, as compared to patients of stage I or II tumors (Figure S2), as expected. Furthermore, patients of prognostic stage II with circ-BAX-18-positive tumors showed an increased probability of poorer DFS (Figure 4A; *p* = 0.003) and OS (Figure 4B; *p* = 0.024), as compared to those with circ-BAX-18-negative tumors of the same prognostic stage.

## 3. Discussion

As BrCa cases and mortality rates continue to rise globally, the epidemiological burden of this disease is steadily worsening. Alongside socioeconomic disparities and various modifiable and non-modifiable risk factors, the profound genetic and molecular heterogeneity of BrCa significantly complicates its clinical management [34]. To address these challenges and decipher the complex molecular basis of the disease, an urgent move towards precision oncology is essential to achieve meaningful improvements in patient outcomes. BrCa guidelines already move beyond conventional pathologic evaluation of breast tumors by integrating essential immunohistochemical markers and commercially available multi-gene panel assays to refine the risk stratification of patients [34]. Therefore, current research must intensify efforts toward the identification and validation of novel, clinically relevant molecular biomarkers to facilitate earlier detection, predict therapeutic responses and ultimately limit the prognostic disparities currently observed across patient cohorts with seemingly identical clinical profiles.

circRNAs are implicated in BrCa progression as their expression shifts across different stages and subtypes of disease [35]. More importantly, they are enriched in the blood and extracellular vesicles of BrCa patients, where they can be quantified as highly stable biomarkers for non-invasive diagnosis and prognostic prediction [36]. circ-BAX-18 is a circular RNA generated through back-splicing of *BAX* pre-mRNA; it is distinguished by the incorporation of a novel microexon with canonical splice sites and is expressed across normal and cancerous breast cell lines [30]. In BrCa, *BAX* mRNA and protein levels have been extensively evaluated, either standalone or in combination with BCL2 (the BAX/BCL2 ratio), to predict prognosis, response to radiation therapy and apoptotic sensitization following treatment with cytotoxic agents [24,25,26,27]. Expanding their clinical utility, here we examine the potential prognostic significance of this novel *BAX*-derived circRNA in a cohort of 144 BrCa patients. For this purpose, we developed and implemented a sensitive qPCR assay using divergent primers, ensuring the accurate and exclusive quantification of circ-BAX-18 over its linear parental mRNA.

Expression analysis revealed a significant downregulation of circ-BAX-18 expression levels in almost all breast tumors compared to their non-cancerous counterparts. However, the relatively small number of paired samples and the observed overlap in expression distributions between cancerous and normal tissues prevent us from establishing a role as a potent diagnostic discriminator. Nevertheless, this dynamic shift in circ-BAX-18 expression during breast oncogenesis merits further investigation. While recent studies document that circRNAs are often globally downregulated in cancer cells, this notion is currently challenged in BrCa [37,38]. Alternatively, the observed circRNA expression is likely driven by the dysregulation of its parental gene [39]. In BrCa, the main *BAX* transcript variant, *BAX-α*, is reportedly downregulated in malignant tissues and cell lines compared to normal epithelium [40,41]. This is consistent with our observations regarding circ-BAX-18 expression. Such downregulation is likely driven by a multifaceted regulatory network, including *BAX* promoter polymorphisms, miRNA-mediated post-transcriptional silencing, and *TP53* impairment [28,42,43]. As BAX represents a key activator of the intrinsic apoptotic pathway, the downregulation of its mRNA and protein levels in BrCa constitutes an essential evolutionary step for malignant cells to evade apoptosis [26,40,44]. Consequently, this suppression of the host gene provides a plausible explanation for the attenuated circ-BAX-18 expression in cancerous tissues compared to adjacent non-cancerous counterparts, likely representing an early event in breast carcinogenesis [45].

Despite the profound overall downregulation of circ-BAX-18 in BrCa, we observed that elevated expression within the patient cohort was a robust indicator of poor prognosis. Collectively, Kaplan–Meier survival analysis and Cox regression models demonstrated that a circ-BAX-18-positive status was associated with a more than 2-fold increase in the risk of tumor recurrence and disease-specific death, independent of other established prognosticators. This finding indicates that in a specific subset of breast tumors, circ-BAX-18 may exert a potential oncogenic role, functioning in contradiction to the tumor-suppressive properties of its parental *BAX* gene [40,46]. Such functional plasticity is not surprising, with ncRNAs frequently acting as a double-edged sword depending on the tumor microenvironment, molecular subtype, and cellular context [47,48,49]. This apparent “paradox” has been observed for other transcripts, as well; for instance, significant downregulation of *HSPB2* mRNA expression occurs in breast tumors compared to their adjacent non-cancerous breast tissues, yet high *HSPB2* mRNA levels are associated with female BC patients’ relapse and poor OS [50]. *BAX*-derived circRNAs are increasingly implicated in the intricate RNA interactome, with recent reports demonstrating their ability to exert oncogenic potential by sequestering specific miRNAs, such as miR-152-5p and miR-4802-5p [30]. Similar antagonistic relationships between circRNAs and their linear counterparts have been documented in other malignancies, involving the *MPP6*/*circMPP6* and *SMARCA5*/*circSMARCA5* regulatory axes [51,52,53]. Such functional diversity is driven by the multifaceted regulatory repertoire of circRNAs. In the nucleus, they can directly modulate the transcription of host genes by interacting with RNA polymerase II or inducing R-loop formation, whereas in the cytoplasm, they exert post-transcriptional control by acting as miRNA sponges or protein decoys [9]. Moreover, investigating the RNA modification landscape and IRES elements is a vital next step. Bridging the translational gap from bench to bedside will require a deeper understanding of how these factors dictate circRNA metabolism and spatial trafficking and how alternative translation of small peptides might impact the post-translational regulation of parental genes [9,54]. In silico annotation of functional elements and regions of circ-BAX-18 unveiled an absence of RNA modification-associated motifs, IRES, and open reading frames (ORFs), suggesting a primary role as a non-coding regulatory RNA rather than a protein-coding template. We hypothesize that circ-BAX-18 acts as a competing endogenous RNA (ceRNA) that sequesters tumor-suppressive miRNAs such as miR-3918 and miR-3663-3p [30]. The sequestration of miR-3918 by circ-BAX-18 could drive FGFR1 overexpression, thus enhancing oncogenic progression in BrCa cells. Moreover, miR-3663-3p is a miRNA with established oncoprotective roles. In gastrointestinal malignancies, miR-3663-3p exerts tumor-suppressive effects by inducing cell cycle arrest, modulating the CCND1 pathway and negatively regulating EGFR/ERK signaling [55,56]. In BrCa, miR-3663-3p expression is significantly modulated following HER2-targeted therapy, suggesting that its depletion through circ-BAX-18-mediated sequestration could serve as a mechanism underlying treatment resistance [57]. This proposed “circRNA–miRNA–mRNA” axis provides a putative biological explanation for BrCa progression and the poorer survival of patients.

More importantly, circ-BAX-18 expression retains its prognostic value within distinct molecular and clinical patient subgroups. Specifically, circ-BAX-18 expression was associated with markedly worse OS in patients with luminal A and TNBC tumors. Luminal A tumors are typically characterized by an indolent disease course with a low but persistent long-term relapse risk. These patients usually benefit from endocrine therapy with tamoxifen and/or aromatase inhibitors, rather than chemotherapy [58,59]. Consequently, molecular profiling is often recommended for patients with luminal A tumors to assess individual relapse risk and determine the administration of adjuvant chemotherapy [59]. Toward this end, circ-BAX-18 assessment could serve as a valuable molecular indicator to identify high-risk luminal A patients who might require long-term monitoring or intensified therapy. Similarly, quantifying circ-BAX-18 ameliorates the risk stratification of patients with TNBC. This is particularly valuable in light of the well-documented “TNBC paradox”, a clinical phenomenon where patients exhibit high response rates to neoadjuvant chemotherapy, yet fail to achieve improved OS, as they remain at an increased risk for early recurrence and metastatic progression [60,61]. Furthermore, circ-BAX-18 positivity enables precise risk stratification among patients with anatomic and prognostic stage II disease, successfully identifying those with a significantly higher risk of relapse and mortality within these heterogeneous subgroups. Therefore, assessing circ-BAX-18 expression levels could serve as a surrogate biomarker to complement prognostic or anatomic staging and molecular subtyping, facilitating the identification of patients at high risk for disease recurrence.

Although our findings support the potential of circ-BAX-18 as a prognostic biomarker in BrCa, several limitations of the present study must be acknowledged. First of all, the retrospective nature of the present single-center study along with the small sample size (*n* = 144) are important limitations *per se*. A notable constraint is also the limited availability of paired non-cancerous tissues (*n* = 14) that were included, limiting our ability to draw strong conclusions on differential expression of circ-BAX-18. An additional limitation of our study is that subgroup analyses were conducted without adjustment for multiple testing; therefore, the corresponding findings could be designated as exploratory. Finally, it is important to note that our study lacks functional validation of circ-BAX-18 in BrCa pathogenesis.

Our clinical findings support the existence of an association between circ-BAX-18 levels and female BrCa patients’ survival. Although a ceRNA-mediated mechanism of circ-BAX-18 could provide a logical basis for our clinical observations, the exact biological role of this circRNA and its involvement in BrCa progression remain to be fully elucidated. To bridge this translational gap, future functional studies are needed to determine the mechanisms underlying expression alteration during breast carcinogenesis.

## 4. Materials and Methods

### 4.1. Cell Culture Conditions

The human BrCa cell line MCF-7 was obtained from the American Tissue Culture Collection (ATCC) (Manassas, VA, USA) and propagated in Dulbecco’s Modified Eagle Medium (Biosera, Cholet, France) containing 2 g/L glucose, 10% FBS, and 1% penicillin/streptomycin, in a humidified incubator (Thermo Fisher Scientific Inc., Carlsbad, CA, USA) at 5% CO_2_, 95% humidity, and 37 °C.

### 4.2. Patients and Tissue Collection

One hundred and forty-four (144) BrCa samples and 14 paired, non-cancerous tissue samples were collected from non-consecutive female patients with primary BrCa, subjected to surgery at the Fourth Department of Surgery, University General Hospital “Attikon”, Athens, Greece. To ensure that the clinical evaluation of circ-BAX-18 expression would not be influenced by unrelated pathologies, we excluded patients presenting with comorbidities at the time of BrCa diagnosis, such as cardiovascular diseases, diabetes, hormonal disorders, or other conditions. A detailed database with biological and clinicopathological data was built; this database included the age of patients, the dimensions of the resected tumor, the infiltration of regional lymph nodes, the presence of distant metastasis, and the histological and molecular subtypes as well as the histological grade of the tumor, the expression status of PR, ER, HER2, and the mitotic rate based on the Ki-67 index. All breast neoplasms were independently characterized by two pathologists. The anatomic (TNM) and prognostic stages were determined based on the collected data, and recorded in the database. All breast tissue specimens were snap-frozen immediately after tumor resection surgery.

The whole original research study was conducted in full accordance with the 1964 Declaration of Helsinki and its later amendments. Our study also received the approval of the institutional Ethics Committee of the University General Hospital “Attikon”, Athens, Greece. All patients provided written informed consent.

### 4.3. Total RNA Extraction and First-Strand cDNA Synthesis

The collected fresh frozen breast tissues were homogenized, and total RNA was then extracted using NucleoZOL (Takara Bio Inc., Shica, Japan). Measurement of the concentration and quality control of the total RNA extracts were carried out with a NanoDrop™ 2000 Spectrophotometer (Thermo Fisher Scientific Inc., Carlsbad, CA, USA), prior to RNA integrity control using agarose gel electrophoresis.

After that, first-strand cDNA synthesis was performed using Maxima™ Reverse Transcriptase (Thermo Fisher Scientific Inc., Carlsbad, CA, USA) and random hexamers, following the manufacturer’s protocol for reverse transcription, starting from 2 µg of total RNA; these reactions were performed in a Veriti™ Thermal Cycler (Thermo Fisher Scientific Inc., Carlsbad, CA, USA).

### 4.4. Quantitative Real-Time PCR (qPCR)

A real-time quantitative PCR (qPCR) assay was applied for the relative quantification of circ-BAX-18 against two housekeeping genes, namely glyceraldehyde-3-phosphate dehydrogenase (*GAPDH*) and hydroxymethylbilane synthase (*HMBS*), which were chosen among others as reference genes, according to the literature data and our previous published results [50]. qPCR assay optimization included designing specific divergent primers for circ-BAX-18 and convergent primers for *GAPDH* and *HMBS* amplification, followed by optimization of primer concentrations. In our study, each reaction was performed in triplicate, to assure the reproducibility of the obtained data. qPCR was performed in a QuantStudio 5 Real-Time PCR System (Thermo Fisher Scientific Inc., Carlsbad, CA, USA), using the KAPA SYBR^®^ FAST qPCR Master Mix (2X) Kit (Kapa Biosystems Inc., Cape Town, South Africa). The sequences of circ-BAX-18 primers were 5′-CTCAAGGCCCTGAGGGA-3′ and 5′-ATGATCTGCTCAGAGCTGGTG-3′; their amplicon length was 85 bp. The sequences of primers for *GAPDH* and *HMBS* cDNA amplification as well as the respective amplicon lengths have previously been described in detail [50]. All primers were used at a final concentration of 200 nM, after optimization, in order to achieve amplification efficiencies very close to 100% and to avoid primer dimer formation. Melt curve analysis confirmed the circRNA-specific amplification in each sample. circ-BAX-18 expression levels were determined with relative quantification [62]. The application of the comparative Ct (2^−∆∆Ct^) method exploited *GAPDH* and *HMBS* as endogenous reference genes to normalize the PCRs for the quantity of RNA added to the reverse transcription, and MCF-7 cDNA as a calibrator sample for rendering results from distinct qPCR runs comparable. The MCF-7 cell line was selected as the calibrator because circ-BAX-18 is expressed more in this cell line than in others, including T-47D, BT-474, BT-20, HCC70, Hs 578T, MDA-MB-231, MDA-MB-453, and MDA-MB-468 [30].

**Figure 5:**
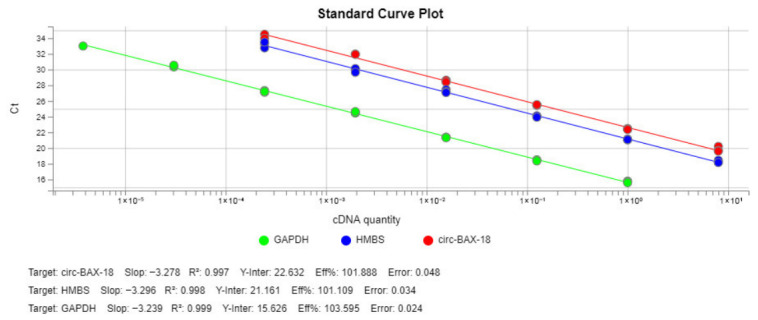
Standard curves of circ-BAX-18, *GAPDH*, and *HMBS* amplification. All three amplification efficiencies are approximately 100%, which is a prerequisite for the application of the comparative Ct (2^−∆∆Ct^) method.

The prerequisites for the application of the 2^−∆∆Ct^ method were checked in a validation experiment, in which the C_t_ values of circ-BAX-18, *GAPDH*, and *HMBS* were measured in a dilution series of MCF-7 cDNA covering several orders of magnitude. The real-time qPCR efficiency (*E*) for each amplicon was calculated using the following formula: *E* = −1 + 10^(−1/α)^, where α is the slope of the corresponding amplification plot. As illustrated in Figure 5, the slopes of the circ-BAX-18, *GAPDH*, and *HMBS* standard curves are very similar; this is indicative of similar amplification efficiencies.

The relative expression of circ-BAX-18 in each sample was determined in RQUs, by calculating the ratio of its levels to the geometric mean of *GAPDH* and *HMBS* transcripts, divided by the same ratio calculated for the calibrator sample. Therefore, our calculations are summarized in the following formula:

$$RQU_{Sample\_X}=\frac{{\left(\frac{C_{circ-BAX-18}}{C_{ref}}\right)}_{Sample\_X}}{{\left(\frac{C_{circ-BAX-18}}{C_{ref}}\right)}_{Calibrator}}$$

where “Sample X” is a random tissue specimen, “C_circ-BAX-18_” is the quantity of circ-BAX-18, and “C_ref_” is the geometric mean of the quantities of *GAPDH* and *HMBS*.

### 4.5. Biostatistical Analysis

circ-BAX-18 expression did not follow a Gaussian distribution in the cohorts of cancerous and non-cancerous breast tissue specimens. Hence, only non-parametric tests (the Mann–Whitney *U* test and Jonckheere–Terpstra test, where appropriate) were applied to assess the significance of differences observed among BrCa patients’ subgroups, generated according to the anatomic stage, the molecular subtype, the histological grade of the tumor, and/or the prognostic stage of the disease. Moreover, the Wilcoxon signed-rank test was applied to assess the significance of differences in circ-BAX-18 expression between the 14 pairs of cancerous and adjacent non-cancerous breast tissues.

Since there is no established cut-off point for circ-BAX-18 expression, we decided to split at the median of the distribution of circ-BAX-18 levels in BrCa samples. This cut-off value was 20.25 RQU. Thus, circ-BAX-18 expression of each sample was classified as high (≥20.25 RQU) or low (<20.25 RQU). Next, we performed chi-square (χ^2^) tests, including Fisher’s exact test where applicable, to examine potential associations between circ-BAX-18 expression status (high vs. low expression) and other categorical variables, such as the ER, PR, and HER2 status, or the mitotic rate based on Ki-67 status.

Survival analysis with regard to DFS and OS was then performed. The significance of differences between the Kaplan–Meier survival curves was assessed using the log-rank (Mantel–Cox) test. The univariate Cox regression included the circ-BAX-18 expression status, the anatomic stage of the disease, the molecular subtype, the histological grade of the tumor, and/or the prognostic stage of the disease. In the multivariate Cox regression, variables were selected based on clinical relevance and a univariate *p* value threshold of <0.10. The prognostic stage was excluded from the same multivariable Cox model as the anatomic stage and molecular subtype to avoid multicollinearity, as these variables share overlapping components. The proportional hazards assumption was checked using residual-based methods to ensure that the hazard ratios for covariates remain constant over time. Bootstrap (1000 random samples) univariate and multivariable Cox regression models were also built; further, the bias-corrected and accelerated (BCa) 95% confidence interval (CI) of each hazard ratio (HR) was calculated. Next, stratified Kaplan–Meier survival analyses were performed in BrCa patients’ subgroups, as patients were stratified according to specific clinicopathological features. Because these subgroup analyses were exploratory, no formal adjustment for multiple testing was performed; hence, results should be interpreted with caution.

Statistical significance was considered in each test when the respective *p* value was calculated to be lower than 0.050.

## 5. Conclusions

In conclusion, higher circ-BAX-18 expression levels appeared to be associated with breast tumor recurrence and poor OS of female BrCa patients. Interestingly, circ-BAX-18 deserves further investigation as a potential surrogate molecular biomarker in BrCa that could add some prognostic information to the prognostic stage of the disease. Moreover, mechanistic experiments are needed to shed light on the potential role of this circRNA in BrCa.

## Figures and Tables

**Table 1:** Biological and clinicopathological features of BrCa patients.

	Number of Patients (%)
**Patients**	144
**circ-BAX-18 expression status**	
Negative	87 (60%)
Positive	57 (40%)
**Age (y)**	Median: 60; Range: 32–90
**Tumor size (cm^2^)**	Median: 2.4; Range: (0.6–8.5)
**Cancer type**	
Invasive ductal carcinoma	117 (81.3%)
Lobular carcinoma	15 (10.4%)
Other carcinomas	12 (8.3%)
**Molecular subtype**	
Luminal A	56 (38.9%)
Luminal B	39 (27.1%)
Triple-negative	34 (23.6%)
HER2-enriched	15 (10.4%)
**Histological grade**	
I	7 (4.9%)
II	93 (64.6%)
III	44 (30.5%)
**HER2 status**	
Negative	110 (79.1%)
Positive	29 (20.9%)
Unknown	5
**ER status**	
Negative	54 (37.5%)
Positive	90 (62.5%)
**PR status**	
Negative	78 (54.2%)
Positive	66 (45.8%)
**Ki-67 index** *	
Low (≤14%)	89 (64.5%)
High (>14%)	49 (35.5%)
Unknown	6
**Anatomic stage**	
I	40 (27.8%)
II	86 (59.7%)
III	18 (12.5%)
**Prognostic stage**	
I	57 (39.6%)
II	66 (45.8%)
III	21 (14.6%)

* Abbreviations: ER, estrogen receptors; PR, progesterone receptors.

**Table 2:** circ-BAX-18 levels in cancerous and non-cancerous breast tissue specimens.

Variable	Mean ± SE	Range	Percentiles		
			25th	50th (Median)	75th
circ-BAX-18 expression (RQU)					
in cancerous tissues (*n* = 144)	29.78 ± 2.43	0.63–149.4	8.51	20.25	40.34
in non-cancerous tissues (*n* = 14)	156.6 ± 38.97	9.84–487.9	40.24	108.9	234.0

Abbreviations: RQU, relative quantification units; SE, standard error.

**Table 3:** Univariate and multivariate Cox regression models predicting the DFS of BrCa patients.

	Univariate Analysis (*n* = 140)					Multivariate Analysis (*n* = 140)				
Covariate	HR	95% CI	*p* Value ^1^	BCa Bootstrap 95% CI	Bootstrap *p* Value ^1^	HR	95% CI	*p* Value ^1^	BCa Bootstrap 95% CI	Bootstrap *p* Value ^1^
circ-BAX-18 expression status										
Negative (*n* = 69)	1.00					1.00				
Positive (*n* = 71)	2.32	1.20–4.49	*0.012*	1.24–4.97	*0.012*	2.46	1.22–4.97	*0.012*	1.19–6.06	*0.011*
Anatomic stage			*0.000*							
I (*n* = 40)	1.00									
II (*n* = 83)	2.09	0.85–5.11	0.11	0.92–8.11	0.088					
III (*n* = 17)	6.43	2.37–17.43	*<0.001*	2.21–33.69	*0.001*					
Molecular subtype			*<0.001*							
Luminal A (*n* = 54)	1.00									
Luminal B (*n* = 38)	1.00	0.36–2.81	1.00	0.28–3.15	0.99					
Triple-negative (*n* = 33)	3.80	1.68–8.60	*0.001*	1.68–12.48	*0.002*					
HER2-enriched (*n* = 15)	5.81	2.34–14.38	*<0.001*	2.39–19.58	*0.001*					
Grade			0.54							
1 (*n* = 7)	1.00									
2 (*n* = 90)	2.01	0.27–14.86	0.49	0.45–26.431	0.23					
3 (*n* = 43)	2.61	0.34–19.76	0.35	0.55–35.640	0.16					
Prognostic stage			*<0.001*					*<0.001*		
I (*n* = 56)	1.00					1.00				
II (*n* = 64)	5.05	1.93–13.25	*0.001*	2.06–22.69	*0.002*	2.56	1.07–6.09	*0.034*	1.14–10.04	*0.022*
III (*n* = 20)	10.64	3.73–30.33	*<0.001*	3.56–66.00	*0.001*	7.54	2.96–19.24	*<0.001*	2.54–38.60	*0.001*

^1^ Statistically significant *p* values are shown in italics. Abbreviations: BCa, bias-corrected and accelerated; CI, confidence interval; HR, hazard ratio.

**Table 4:** Univariate and multivariate Cox regression models predicting the OS of BrCa patients.

Covariate	Univariate Analysis (*n* = 140)					Multivariate Analysis (*n* = 140)				
	HR	95% CI	*p* Value ^1^	BCa bootstrap 95% CI	Bootstrap *p* Value ^1^	HR	95% CI	*p* Value ^1^	BCa Bootstrap 95% CI	Bootstrap *p* Value ^1^
circ-BAX-18 expression status										
Negative (*n* = 69)	1.00					1.00				
Positive (*n* = 71)	2.38	1.18–4.81	*0.015*	1.25–5.46	*0.015*	2.45	1.26–4.74	*0.008*	1.23–5.60	*0.011*
Anatomic stage			*0.001*							
I (*n* = 40)	1.00									
II (*n* = 83)	1.91	0.77–4.70	0.16	0.62–10,980	0.16					
III (*n* = 17)	5.75	2.09–15.84	*0.001*	1.52–401.63	*0.001*					
Molecular subtype			*0.001*							
Luminal A (*n* = 54)	1.00									
Luminal B (*n* = 38)	1.12	0.39–3.24	0.83	0.38–3.31	0.84					
Triple-negative (*n* = 33)	4.56	1.98–10.49	*<0.001*	1.94–15.64	*0.001*					
HER2-enriched (*n* = 15)	3.38	1.17–9.75	*0.024*	0.96–12.51	*0.017*					
Grade			0.76							
1 (*n* = 7)	1.00									
2 (*n* = 90)	0.82	0.19–3.46	0.78	0.15–24.173	0.66					
3 (*n* = 43)	1.05	0.24–4.65	0.95	0.18–28.170	0.96					
Prognostic stage			*<0.001*					*<0.001*		
I (*n* = 56)	1.00					1.00				
II (*n* = 64)	2.62	1.10–6.24	*0.029*	1.18–8.15	*0.015*	5.03	1.91–13.20	*0.001*	1.74–233.42	*0.002*
III (*n* = 20)	7.27	2.85–18.52	*<0.001*	2.67–26.83	*0.001*	11.36	3.98–32.49	*<0.001*	2.82–107.18	*0.002*

^1^ Statistically significant *p* values are shown in italics. Abbreviations: BCa, bias-corrected and accelerated; CI, confidence interval; HR, hazard ratio.

## Data Availability

The data presented in this study are available on reasonable request from the corresponding author.

## Supplementary Materials

The following supporting information can be downloaded at https://www.mdpi.com/article/10.3390/ijms27104160/s1.
