# Supplementary material for: Upregulation of a Circular BAX Transcript in Breast Cancer Is Associated with Unfavorable Prognosis
Source: Int J Mol Sci. 2026 May 7;27(10):4160. doi: 10.3390/ijms27104160 (PMC13206390; doi:10.3390/ijms27104160)
Supplement: Supplementary file 1 [file ijms-27-04160-s001.zip › Table S1.pdf]

Table S1. Multivariate Cox regression predicting the DFS of BrCa patients.

	Multivariate analysis (*n*=140)				
**Covariate**	**HR**	**95% CI**	***P* value [1]**	**BCa bootstrap 95% CI**	**Bootstrap *P* value [1]**
circ-BAX-18 expression status					
Negative (*n*=69)	1.00				
Positive (*n*=71)	3.23	1.58 – 6.59	*0.001*	1.56 – 8.59	*0.002*
Anatomic stage			*0.001*		
I (*n*=40)	1.00				
II (*n*=83)	1.21	0.48 – 3.06	0.68	0.28 – 41.018	0.67
III (*n*=17)	4.83	1.69 – 13.85	*0.003*	1.16 – 181.183	*0.001*
Molecular subtype			*<0.001*		
Luminal A (*n*=54)	1.00				
Luminal B (*n*=38)	1.47	0.50 – 4.34	0.48	0.50 – 3.98	0.54
Triple-negative (*n*=33)	4.18	1.77 – 9.86	*0.001*	1.64 – 12.41	*0.001*
HER2-enriched (*n*=15)	6.76	2.62 – 17.48	*<0.001*	2.28 – 26.01	*0.001*

[1] Statistically significant *P* values are shown in italics.
Abbreviations: BCa, bias-corrected and accelerated; CI, confidence interval; HR, hazard ratio.
