# Supplementary material for: Upregulation of a Circular BAX Transcript in Breast Cancer Is Associated with Unfavorable Prognosis
Source: Int J Mol Sci. 2026 May 7;27(10):4160. doi: 10.3390/ijms27104160 (PMC13206390; doi:10.3390/ijms27104160)
Supplement: Supplementary file 1 [file ijms-27-04160-s001.zip › Table S2.pdf]

Table S2. Multivariate Cox regression predicting the OS of BrCa patients.

	Multivariate analysis (*n*=140)				
**Covariate**	**HR**	**95% CI**	***P* value [1]**	**BCa bootstrap 95% CI**	**Bootstrap *P* value [1]**
circ-BAX-18 expression status					
Negative (*n*=69)	1.00				
Positive (*n*=71)	2.85	1.39 – 5.87	*0.004*	1.24 – 8.95	*0.006*
Anatomic stage			*0.007*		
I (*n*=40)	1.00				
II (*n*=83)	1.24	0.49 – 3.15	0.65	0.39 – 6.22	0.67
III (*n*=17)	4.04	1.38 – 11.85	*0.011*	1.08 – 34.46	*0.012*
Molecular subtype			*0.003*		
Luminal A (*n*=54)	1.00				
Luminal B (*n*=38)	1.49	0.50 – 4.42	0.48	0.35 – 6.36	0.48
Triple-negative (*n*=33)	4.42	1.88 – 10.42	*0.001*	1.75 – 16.18	*0.001*
HER2-enriched (*n*=15)	3.62	1.24 – 10.57	*0.018*	0.76 – 20.49	*0.033*

[1] Statistically significant *P* values are shown in italics.
Abbreviations: BCa, bias-corrected and accelerated; CI, confidence interval; HR, hazard ratio.
